# VEGF-mediated tight junctions pathological fenestration enhances doxorubicin-loaded glycolipid-like nanoparticles traversing BBB for glioblastoma-targeting therapy

**DOI:** 10.1080/10717544.2017.1386731

**Published:** 2017-11-28

**Authors:** Lijuan Wen, Yanan Tan, Suhuan Dai, Yun Zhu, Tingting Meng, Xiqin Yang, Yupeng Liu, Xuan Liu, Hong Yuan, Fuqiang Hu

**Affiliations:** a College of Pharmaceutical Science, Zhejiang University, Hangzhou, Zhejiang, People’s Republic of China;; b Ocean College, Zhejiang University, Zhoushan, Zhejiang, People’s Republic of China

**Keywords:** VEGF, glioblastoma, pathological, tight junctions, fenestration

## Abstract

The existence of blood–brain barrier (BBB) greatly hindered the penetration and accumulation of chemotherapeutics into glioblastoma (GBM), accompany with poor therapeutic effects. The growth of GBM supervene the impairment of tight junctions (TJs); however, the pathogenesis of BBB breakdown in GBM is essentially poorly understood. This study found that vascular endothelial growth factor (VEGF) secreted by GBM cells plays an important role in increasing the permeability of BBB by disrupting endothelial tight junction proteins claudin-5 and thus gave doxorubicin (DOX)-loaded glycolipid-like nanoparticles (Ap-CSSA/DOX), an effective entrance to brain tumor region for GBM-targeting therapy. In addition, VEGF downregulates the expression of claudin-5 with a dose-dependent mode, and interfering with the VEGF/VEGFR pathway using its inhibitor axitinib could reduce the permeability of BBB and enhance the integrity of the barrier. Ap-CSSA/DOX nanoparticles showed high affinity to expressed low-density lipoprotein receptor-related proteins 1 (LRP1) in both BBB and GBM. And BBB pathological fenestration in GBM further exposed more LRP1 binding sites for Ap-CSSA/DOX nanoparticles targeting to brain tumor, resulting in a higher transmembrane transport ratio *in vitro* and a stronger brain tumor biodistribution *in vivo*, and finally realizing a considerable antitumor effect. Overall, taking advantage of BBB pathological features to design an appropriate nanodrug delivery system (NDDS) might provide new insights into other central nervous system (CNS) diseases treatment.

## Introduction

Blood–brain barrier (BBB) is a unique system that supports and protects the function of central nervous system (CNS) (Tajes et al., 2014). It is a specialized system of capillary endothelial cells that are partially covered by pericytes and basement membrane and mostly fully surrounded by the end of astrocytes (Wei et al., 2014a). These brain endothelial cells lack fenestration and exhibit extremely low rates of pinocytosis/transcytosis and are also involved in the regulation of BBB permeability (Huang et al., 2011b; Daneman, 2012). Electron microscopy (EM) studies with ferritin and horseradish peroxidase have since shown that the BBB is localized at the level of tight junctions (TJs) between adjacent brain endothelial cells (Zlokovic, 2008). Further, it is known that in several areas of the brain BBB is very thin or supposed to be loose or weak, and these are identified in Pineal body, neurohypophysis and area postrema (Upadhyay, 2014). The barrier restricts the free leakage of most large molecules and more than 98% of small-molecule pharmaceuticals, being a nightmare for diagnosis and therapy of CNS diseases (Juillerat-Jeanneret, 2008; Wei et al., 2015). TJs between endothelial cells in BBB also make the barrier 50–100 times tighter than peripheral microvessels (Zlokovic, 2008). The claudin family is essential in the formation of TJs and plays a key role in the appearance of BBB properties (Tsukita & Furuse, 2002; Wolburg et al., 2002). Loss of claudin proteins is closely associated with the disturbance of BBB function (Jiao et al., 2015). BBB integrity disruption and pathological dysfunction are observed in many CNS disorders, including glioblastoma (GBM) (Schneider et al., 2004), as well as multiple sclerosis (Kirk et al., 2003), Alzheimer’s disease (Claudio, 1995) and ischemic (Xiao et al., 2017), so the BBB itself is a therapeutic target.

Different from normal brain capillaries, the TJs between endothelial cells are damaged because of the growing of GBM, then resulting in the pathological fenestration and leakage of the BBB (Breier et al., 1998). However, the pathogenesis of BBB breakdown in GBM is essentially poorly understood. Vascular endothelial growth factor (VEGF) is originally discovered as a vascular permeability factor and is required for the normal development of embryonic vascular system (Greenberg & Jin, 2005; Jain et al., 2007). VEGF-signaling pathway plays an important role in GBM growth, metabolism and metastasis (Huang et al., 2013). Interestingly, studies have confirmed that VEGF is an important factor in destructing the BBB in autoimmune encephalomyelitis (Argaw et al., 2009) and leukemia (Feng et al., 2011). The action of VEGF/VEGFR signal loops also enhance the disruption of BBB (Lafuente et al., 2006). In this study, VEGF was involved in the mechanism study of TJs pathological opening and BBB pathological leakage. Taking advantage of pathological features of the BBB to design a feasible nanodrug delivery system (NDDS) may provide new insights into brain tumor effective therapy.

Nontargeting drug delivery systems were limited in clinical treatment because of their lack of specific selectivity to cross the BBB and then reach to brain tumor (Martíncarbonero et al., 2008). So making full use of BBB pathological characteristics to design an appropriate targeting nano drug delivery system (TNDDS) for GBM effective treatment is imperative. Low-density lipoprotein receptor-related proteins 1 (LRP1), a member of low-density lipoprotein (LDLR) family, is highly expressed at the BBB and also overexpressed in GBM cells (Maletínská et al., 2000; Ng et al., 2016). BBB pathological opening in GBM would expose more LRP1-binding sites, making LRP1 a potential receptor for brain tumor-targeting therapy. Angiopep-2 (Ap) is derived from the Kunitz protease domain of aprotinin, exhibiting a high affinity to LRP1 (Yamamoto et al., 1997; Wei et al., 2014b). Here, we chose Ap as a specific ligand to decorate on the surface of copolymer micelles via a PEG liker for BBB targeting and GBM cascade targeting.

In this study, we constructed an Ap-conjugated glycolipid-like drug delivery system (Ap-CSSA/DOX), modified with Ap for BBB targeting, as well as brain tumor cells targeting and quickly internalization. Doxorubicin (DOX), a broad-spectrum antitumor drug, could not cross the BBB to reach brain tumor (Wang et al., 2015). Its antitumor efficiency is extremely hampered by its serious side effect especially cardiotoxicity (Chen et al., 2015). Herein, DOX was physically encapsulated into the hydrophobic core of Ap-CSSA copolymer micelles. *In vitro* coculture model and monolayer model were used to simulate pathological BBB and physiological BBB, respectively. GBM growth resulting in BBB pathological fenestration was detected *in vitro* and *in vivo*. TJs pathological fenestration enhancing Ap-CSSA/DOX nanoparticles crossing the BBB and then cascade-targeting GBM was also conducted. Immunofluorescence of claudin-5 assay was to explore the molecular biology mechanisms of BBB pathological opening, and the relationship between VEGF/VEGFR signal pathway and the expression of claudin-5 were also verified. Furthermore, both the competition biodistribution assays *in vivo* and cellular competition uptake assays *in vitro* were used to assess the pathway of Ap-CSSA copolymer micelles for GBM targeting.

## Materials and methods

### Materials

Chitosan oligosaccharide (CSO) with average molecular weight of 18.0 kDa was prepared by enzymatic degradation of chitosan (CS) (95% acetylate, *M*_w_ = 450 kDa; Yuhuan Marine Biochemistry Co., Ltd, Zhejiang, China) as described in a previous work (Zhao et al., 2012; Yi et al., 2015). Angiopep-2 peptide (Ap, TFFYGGSRGKRNNFKTEEY) was synthesized by Chinese Peptide Company (Hangzhou, China). NH_2_-PEG-NH_2_ (PEG_2000_) and 2, 4, 6-trinitrobenzene sulfonic acid (TNBS) were purchased from Sigma-Aldrich (Diegem, Belgium). Pyrene was purchased from Aldrich Chemical Co (Milwaukee, WI). Di-tert-butyl dicarbonate ((Boc)_2 _O), 1-ethyl-3-(3-dimethyl-aminopropyl) carbodiimide hydrochloride (EDC) and N-hydroxysuccinimide (NHS) were purchased from Shanghai Medpep Co, Ltd (Pudong, Shanghai, China). N,N′-Disuccinimidyl carbonate (DSC) was obtained from Bio Basic Inc., (Toronto, Canada). Doxorubicin hydrochloride (DOX·HCl) was purchased from Zhejiang Hisun Pharmaceutical Co, Ltd, (Taizhou, Zhejiang, China). 1, 1′-Dioctadecyl-3, 3, 3′, 3′-tetramethylindotricarbocyanine iodide (DiR) was purchased from Life Technologies (Carlsbad, CA). D-Luciferin was supplied by Shanghai Sciencelight Biology Science and Technology Co., Ltd (Shanghai, China). All of the other chemicals were of analytical or chromatographic grade.

### Cell culture

U87 MG cells were cultured at 37 °C in a humidified atmosphere containing 5% CO_2_ in α-MEM (Gibco, Merelbeke, Belgium) supplemented with 10% fetal bovine serum, 100 IU/mL penicillin–streptomycin. bEnd.3 cells were cultured in Dulbecco’s minimum essential medium (DMEM) purchased from Gibco (Merelbeke, Belgium) supplemented with 10% fetal bovine serum (FBS) (Gibco, Billings, MT), 100 IU/mL streptomycin and 100 IU/mL penicillin. The cells were subcultured regularly using trypsin/ethylene diamine tetraacetic acid (EDTA).

### Orthotopic GBM model

Male BALB/C nude mice (18–20 g) were purchased from the Shanghai Silaike Laboratory Animal Limited Liability Company. The U87-Luci orthotopic tumor model was established by injection of U87-luci cells (5 × 10^5^ cells in 10 μL of serum-free culture medium) into the right striatum of each male nude mice (2 mm lateral to the bregma, 0.8 mm anterior to the bregma and 3 mm deep from the dura) at a rate of 1.0 μL/min using a stereotaxic apparatus equipped with a mouse adapter (RWD Life Science Co., Ltd, Shenzhen, China). All of the animal procedures were performed according to national regulations and protocols approved by the ethical committee of Zhejiang University.

### Synthesis and characteristics of Ap-modifiedglycolipid-like copolymers

Glycolipid-like copolymers (CSSA) was synthesized by the method as described before (Liu et al., 2016). In order to synthesis Ap-modified glycolipid-like copolymers (Ap-CSSA), 30 mg Ap and 36 μL di-tert-butyl dicarbonate (Boc)_2 _O was dissolved in dried dimethyl sulfoxide, the reaction was performed at room temperature for 12 h in dark to protect the amine group of peptide. Then, the mixture was activated by prescription dosage 1-ethyl-3-(3-dimethyl-aminopropyl) carbodiimide hydrochloride and N-hydroxysuccinimide (EDC/NHS) for 90 min, 27 mg PEG_2000_ was added and stirred for another 24 h. After that, N, N′-disuccinimidyl carbonate (DSC) was added, the reaction solution was then added into CSSA dissolved in deionized (DI) water and stirred for 24 h. Then, HCl was added to remove the protection of (Boc)_2 _O to Ap. Later, the pH of the reaction solution was adjusted to neutral. The final production was received by lyophilization after the reaction solution dialyzing against DI water for two days.

The ^1^HNMR spectra of CSSA and Ap-CSSA copolymers were measured by ^1^HNMR spectrometer (AC-80, BrukerBiospin, Germany) in D_2_O. The amino-substitution degrees (SD) of CSSA and Ap-CSSA copolymers were determined by 2,4,6-trinitrobenzene sulfonic acid (TNBS) test (Liu et al., 2016). Pyrene was used as a probe to estimate the critical micelle concentrations (CMC) and a Zetasizer (3000HS; Malvern Instruments Ltd, Worcestershire, UK) was used to measure the size and zeta potential of copolymer micelles. The morphological characterization of copolymer micelles samples staining with 2.0% (w/v) phosphotungstic acid were observed by transmission electron microscopy (TEM, JEM-1230, JEOL).

### Preparation and characterization of DOX-loaded glycolipid-like nanoparticles

Doxorubicin (DOX) was obtained by the reaction of doxorucibin hydrochloride with triethylamine in DMSO at room temperature for 12 h as described in reported article (Meng et al., 2016). DOX-loaded Ap-modified glycolipid-like nanoparticles (Ap-CSSA/DOX) were prepared by dialysis method. Briefly, DOX was dissolved in DMSO and Ap-CSSA copolymer was dissolved in DI water. Then, DOX/DMSO solution was added dropwise into Ap-CSSA solution with constant stirring at room temperature for 2 h under light protection. The solution was dialyzed against DI water and then centrifugated at 8000 rpm for 10 min to remove unloaded DOX. CSSA/DOX nanoparticles were prepared by the same method. The size and zeta potential, the morphological characterizations of DOX-loaded nanoparticles were all obtained with the same methods as described above. The drug loading and encapsulation efficiency values of DOX-loaded nanoparticles were measured by the centrifugal–ultrafiltration method. Fluorescence spectrophotometer (F-2500; Hitachi Co, Tokyo, Japan) was used to measure the fluorescent intensity of the DOX.

The drug loading (DL) and encapsulation efficiency (EE) values of DOX-loaded nanoparticles were calculated by the following equations:

(1) DL%= [weight of DOX in nanoparticles/weight of DOX−loaded nanoparticles]×100%

(2) EE%=[weight of DOX in nanoparticles/weight of DOX in feed]×100%

*In vitro* release profiles of DOX were performed by dialyzing the DOX-loaded nanoparticles suspension into 30 mL of phosphate buffer saline (PBS, pH 7.4 and pH 6.5) at 37 °C under continuously shaking (100 rpm) for 48 h. The PBS buffer was replaced at predetermined time intervals and the concentration of DOX in the samples was measured by Fluorescence spectrophotometer.

### Cytotoxicity

The cell cytotoxicity of DOX-loaded nanoparticles were performed by MTT assay. Briefly, U87 MG cells were seeded into 96-well plates at a density of 8 × 10^3^ cells per well in 200 μL of complete medium. After 12-h incubation, the cells were exposed to serial concentrations of DOX-loaded nanoparticles (DOX were in the range of 0–10 μg/mL) at 37 °C for 48 h. At the end of incubation, 20 μL of MTT solution (5 mg/mL in DI water) was added into each well and incubated at 37 °C for another 4 h, then the medium was replaced with 200 μL DMSO to dissolve the purple formazan crystals. After shaking for 15 min, the absorbance of each well was measured by a micro plate reader (Model 680; BioRad, Hercules, CA) at 570 nm. The results demonstrated the cytotoxicity of different DOX formulations. The cell viability of blank CSSA or Ap-CSSA copolymer micelles on bEnd.3 cells and U87 MG cells was determined by the same method.

### Cellular uptake

bEnd.3 cells or U87 MG cells were seeded into a 24-well plates at a density of 5 × 10^4^ cells/cell at 37 °Cand incubated in 5% CO_2_ for 12 h to attach. The cells were then incubated with FITC-labeled CSSA and Ap-CSSA copolymer micelles with equivalent concentration at 100 μg/mL for 1 h, 4 h at 37 °C. Confocal laser scanning microscopy (CLSM, Olympus IX81-FV1000, Japan) was used to observe the cells with flow cytometry for the quantitative analysis of cellular uptake.

### Three-dimensional tumor spheroid uptake

U87 MG cells were seeded into 96-well plates pre-coated with 50 μL low melting point sterile agarose solution (15%, w/v) at a density of 4 × 10^3^ cells per well and incubated at 37 °C. After the spheroids reached a uniform size with uniform and compact structure, tumor spheroids were treated with Ap-CSSA/DOX nanoparticles, CSSA/DOX nanoparticles and doxorucibin hydrochloride with a concentration of DOX at 1.5 μg/mL for 1 h at 37 °C. The spheroids were final subjected to CLSM on an *x*–*z* scanning mode. Semiquantitative fluorescence intensity was then calculated by Image J Software (Bethesda, MD, USA). 

### Cellular internalization of Ap-modified glycolipid-like copolymer micelles

The attached bEnd.3 cells or U87 MG cells were incubated with CSSA/DOX and Ap-CSSA/DOX nanoparticles with equivalent concentration of DOX at 1.5 μg/mL for 6 h at 37 °C. Then, the PBS washed cells were blocked with 3% BSA for 30 min at room temperature. The cells were then incubated with anti-LRP1 primary antibodies (1:50 dilution; Abclonal) for 12 h at 4 °C, The coverslips were finally placed on glass slides, sealed and observed by CLSM.

For the internalization routes of Ap-CSSA copolymer micelles, U87 MG cells were pre-incubated with serum-free culture medium added with various endocytosis inhibitors for 30 min, including chlorpromazine hydrochloride (10 μg/mL), 0.45 M sucrose, cytochalasin D (5 μM), 5-(N-ethyl-N-isopropyl) amiloride (EIPA) (100 μM), 30 μM nystatin and 200 μg/mL Ap. Then incubated with FITC-labeled Ap-CSSA copolymer micelles for another 1 h.

### TJs pathological disruption

Claudin-5 plays a key role in the formation of TJs, and here, we considered it as a marker of TJs pathological breakdown and BBB pathological disruption. The *in vitro* physiological BBB model was constructed according the literature reported (Chen et al., 2014). The bEnd.3 cells were seeded on polycarbonate filter membranes with a pore size of 0.4 μm and a surface area of 1.12 cm^2^ (Costar Transwell, Millipore Corp., Bedford, MA) at a density of 10 × 10^4^ cells per filter. During the culture for 15 days, the medium in both upper and lower compartments were changed every other days, and the integrity of cell monolayer was verified by measuring the transepithelial electrical resistances (TEER >150 Ωcm^2^) values using a Millicell-ERS volt-ohmmeter (Millipore Co., MA, USA). A bEnd.3 cells and U87 MG cells cocultured model was established to imitate a pathological BBB (Lin et al., 2016). U87 MG cells were seeded into 12-well plates at a density of 15 × 10^4^ cells/cell at 37 °C for 12 h to attach, then the inserts with monolayer bEnd.3 cells with feasible TEER were transferred to the plates with confluent U87 MG cells. The two kinds of cells in the transwell-chambers were coculture for another 24 h. The polycarbonate membranes covered by the bEnd.3 cells monolayer were carefully separated from the transwell inserts, washed with PBS and fixed with 4% formaldehyde for 15 min, then blocked with 10% BSA for 30 min at room temperature. The cells were then incubated with anti-claudin-5 primary antibodies (1:40 dilution; Abcam) overnight at 4 °C, followed by incubation with anti-rabbit second antibodies.

Ectogenic VEGF and its signal pathway inhibitor axitinib were used to further verify the effect of VEGF/VEGFR-signaling pathway on the expression of claudin-5. Human VEGF (Peprotech, Rocky Hill, NJ) was added at a concentration of 100 ng/mL, and VEGF-signaling interfering assay with axitinib reached a final concentration at 0 μg/mL, 2 μg/mL and 4 μg/mL.

In order to further observe the claudin-5 change under pathological condition *in vivo*, brain sections were stained with rabbit anti-mouse anti-claudin-5 antibody and near-infrared fluorescent marker-labeled anti-rabbit secondary antibody. Nuclear was stained with 0.5 μg/mL of DAPI. Then, the fluorescent distribution was determined via CLSM on an x-y-z scanning mode.

### Transport of ap-CSSA/DOX nanoparticles crossing pathological and physiological BBB *in vitro*

The *in vitro* pathological BBB model were constructed as described above. Then, bEnd.3 cells monolayer grown on porous transwell insert membranes were incubated with DOX-loaded nanoparticles for 1 h and 4 h. For qualitative analysis, the inserts were moved away at a certain time, and the fixed U87 MG cells were observed under a CLSM.

The quantitative transport of DOX-loaded nanoparticles in pathological BBB model and physiological BBB model was also conducted. At the predetermined time, the amount of DOX in the basolateral medium and cellular uptaken were extracted and then measured by Fluorescence spectrophotometer. The cumulative transfer capacity was calculated as the sum of DOX-loaded nanoparticles quantified at each time point.

### Ultrastructure analysis of TJs *in vivo*

The ultrastructure of brain microvascular endothelial cells was determined by transmission electron microscopy (TEM). Normal brain corpus striatum tissues, tumor tissues and tumor border tissues were obtained and then incised into 1 × 1×1 mm^3^ scraps, followed by immersing into a 2.5% glutaraldehyde solution. The scraps were washed with PBS (pH 7.4) and then stained with 1% osmic acid. After gradient alcohol dehydration, the scraps were embedded in epoxy resin and then cut into ultrathin sections with an ultramicrotome (Wang et al., 2007). The ultra-structure was stained with uranyl acetate and lead citrate, and then observed under TEM.

### *In vivo* bio-distribution of Ap-CSSA copolymer micelles in normal model and tumor-bearing model

Near infrared dye DiR was physically encapsulated into glycolipid-like copolymer micelles to keep track of micelles *in vivo* (Xiang et al., 2011). Ap-CSSA/DiR micelles were prepared with the same method as reported literatures (Hu et al., 2015). To investigate the distribution discrepancy of Ap-CSSA copolymer micelles in pathological BBB and physiological BBB, *in vivo* imaging analysis was also conducted in normal mice. Ten days after tumor implantation, Ap-CSSA/DiR and CSSA/DiR were injected into the tail vein of tumor-bearing nude mice, and the normal nude mice were served as control. Image were taken by Maestro *in vivo* Imaging System (CRI, Woburn, MA), and brain tissues were excised for *ex vivo* fluorescence imaging.

*In vivo* competition assay was performed to confirm the pathway of Ap-CSSA copolymer micelles uptake into the tumor-bearing brains of mice. Briefly, angiopep-2 (10 mg/kg) dissolved in saline was given to mice via the tail vein 30 min in advance, Ap-CSSA/DiR were intravenously administrated. The fluorescent images *in vivo* and *ex vivo* were then obtained.

### *In vivo* antitumor activity

Orthotropic tumor-bearing nude mice were established as describe above. The mice were then randomly divided into four groups: saline group, DOX·HCl group, Ap-CSSA/DOX group and CSSA/DOX group. Each mouse received a dose of 2 mg DOX/kg via the tail vein on days 5, 8 and 11 postinoculation. Mice were imaged noninvasively for luciferase expression 2 days after each injection (days 4, 7, 10, 13). Bioluminescence imaging was obtained using bioluminescence imaging modality (IVIS Spectrum) to mirror the GBM growth response. The quantitative total bioluminescence was measured by drawing regions of interest (ROIs) around tumor areas enclosing emitted signals (*n* = 3). One day after the treatment, three mice from each group were sacrificed, brains and hearts were harvested and then sampled and subjected to routine hematoxylin and eosin (H&E) staining. Body weights were measured daily after implantation (*n* = 6). Survival time of tumor-bearing mice was also recorded (*n*= 6) by using log-rank test in the Kaplan–Meier analysis method.

### *In vivo* distribution of DOX-loaded glycolipid-like nanoparticles in tumor tissue

Ap-CSSA/DOX and CSSA/DOX nanoparticles were intravenously injected in tumor-bearing mice at a dose of DOX at 2 mg/kg. About 4 h later, animals were anesthetized. Brain tissues were removed, subjected to O.C.T.embedding medium and finally frozen at −80 °C. Frozen sections were then observed with a confocal microscope.

### Statistical analysis

Analysis was determined by the computer program GraghPad Prism software (San Diego, CA, USA). Data of all results were expressed as means ± standard deviations (SD) with at least three independent experiments, and the comparison was calculated by Student’s *t*-test. The statistical significance was defined as a *p* value less than 0.05. *p < .05, **p < .01, ***p < .001 were performed in one way ANOVA (*t*-test).

## Results

### Preparation and characterization of Ap-CSSA copolymer micelles

**Figure 1.**
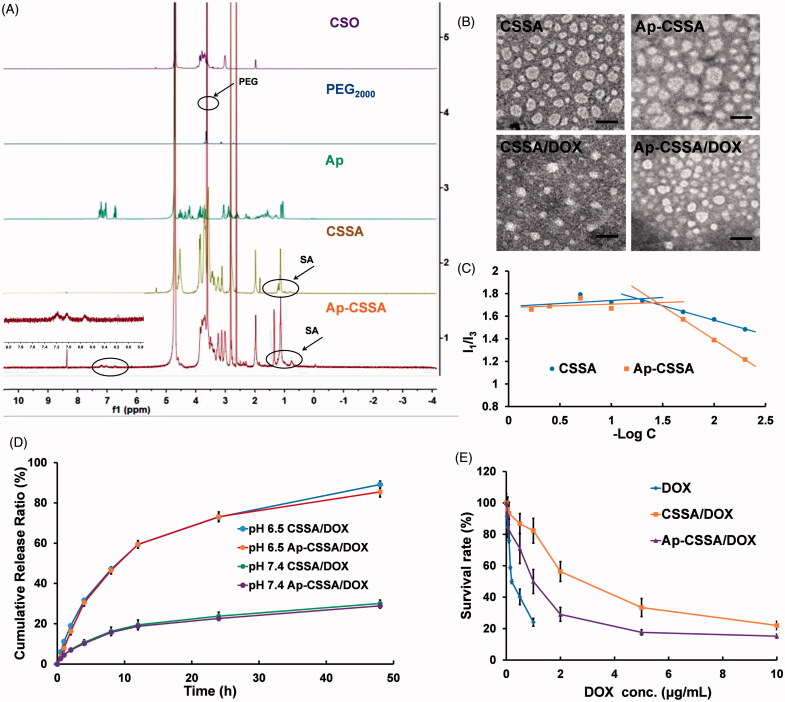
Characterization of glycolipid-like copolymer micelles. (A) ^1^HNMR spectra of CSO, PEG_2000_, Ap, CSSA and Ap-CSSA in D_2_O. (B) TEM images of blank vehicles and DOX-loaded nanoparticles (Scale bar =50 nm). (C) The pyrene fluorescence intensity ratio of *I*_1_/*I*_3_ as a function of the logarithmic concentration (−Log C) of CSSA and Ap-CSSA copolymer micelles. (D) *In vitro* DOX release behaviors of CSSA/DOX and Ap-CSSA/DOX nanoparticles in PBS of pH 7.4 and pH 6.5 (*n* = 3). (E) DOX, CSSA/DOX nanoparticles and Ap-CSSA/DOX nanoparticles against U87 MG with a concentration of 0–10 μg DOX-equiv./mL for 48 h. (*n* = 5).

CSSA copolymer was firstly obtained by the reaction between amino groups of chitosan oligosaccharide (CSO) and carboxyl group of stearic acid (SA) using EDC as the coupling reagent. t-Boc-Ap-PEG-NH_2_ was synthesized by the reaction between carboxyl group of Ap and amino groups of NH_2_-PEG-NH_2_ in the presence of EDC/NHS, while amino terminus of Ap was pre-protected by (Boc)_2 _O. Then, the t-Boc-Ap-PEG-CSSA was obtained by conjugating the remaining primary amino groups in CSSA copolymer with the remaining amino groups of NH_2_-PEG-NH_2_ in the presence of DSC. ^1^H NMR spectrum in D_2_O showed the structure of Ap-CSSA copolymer (Figure 1(A)). The chemical shift at 1.09 ppm and 1.12 ppm was attributed to the methylene hydrogen and methyl hydrogen of SA, respectively. The intense peaks between 3.4 ppm and 3.6 ppm belonged to the methylene hydrogen of NH_2_-PEG-NH_2_. The tiny waves near peaks at 6.75 ppm, 7.05 ppm and 7.20 ppm were attributed to amino acids of benzene from Ap. The results implied that Ap was successfully conjugated with CSSA copolymer. The amino-substitution degree (SD %) of CSSA copolymer (9.57%) and Ap-CSSA copolymer (10.5%) indicated that every 10 glucosamine sugar moiety in CSO polymer shared an Ap molecular modification.

The average sizes of CSSA and Ap-CSSA copolymer micelles were determined as 35.7 ± 1.5 nm and 39.9 ± 2.1 nm, respectively (Table S1). Also, zeta potential of Ap-CSSA was measured as 19.8 ± 0.5 mV, which was lower than that of CSSA (25.5 ± 0.1 mV) (Table S1). These results might be attributed to the stretch of PEG chains and the reduced amino groups after Ap conjugation. Both CSSA and Ap-CSSA copolymer micelles exhibited spherical morphologies (Figure 1(B)). In addition, both CSSA and Ap-CSSA copolymers could self-assemble into nano-scaled micelles due to the hydrophobic SA modification on water-soluble CSO. Figure 1(C) showed the variation of *I*_1_/*I*_3_ ratio against the logarithmic concentration (−Log C) of CSSA and Ap-CSSA copolymer micelles. The elongated hydrophilic chains of PEG and Ap modification induced the CMC of blank vehicles increased from 18.56 μg/mL (CSSA) to 27.98 μg/mL (Ap-CSSA).

### Preparation and characterization of DOX-loaded nanoparticles

As shown in Table S1, compared with CSSA/DOX nanoparticles (7.48 ± 0.03% and 80.90 ± 0.29%), the drug-loading value (6.84 ± 0.06%) and the encapsulation efficiency (73.37 ± 0.63%) of Ap-CSSA/DOX nanoparticles slightly decreased, which was mainly on account of the decoration of Ap and PEG. Since the cohesive force of the hydrophobic interaction between DOX and the hydrophobic cores of micelles was enhanced after drug loading, the particle size of CSSA/DOX nanoparticles (28.6 ± 3.1 nm) and Ap-CSSA/DOX nanoparticles (33.5 ± 4.2 nm) displayed smaller than their blank micelles. Figure 1(B) illustrated that CSSA/DOX and Ap-CSSA/DOX nanoparticles were also spherical morphologies.

Drug release behavior was evaluated in pH 7.4 and pH 6.5 PBS so as to mimic the *in vivo* biological environment and the milieu of tumor, respectively (Li et al., 2016; Liu et al., 2017). With the time prolonged, the accumulative release percentage of CSSA/DOX and Ap-CSSA/DOX nanoparticles in pH 6.5 reached 89.1% and 85.4%, respectively. Interestingly, slower and limited release of DOX from both CSSA/DOX (30.1%) and Ap-CSSA/DOX (28.9%) nanoparticles was noted in neutral condition (Figure 1(D)). The phenomenon was favorable for Ap-CSSA/DOX nanoparticles, since large amounts of drugs will be released rapidly in the acidic environments of tumor.

### Cytotoxicity assay

MTT assay was used to evaluate the safety of blank vehicles and investigate the cell cytotoxicity of DOX-loaded nanoparticles. As shown in Figure S1, cells viability percentage remained almost 80% even when the concentration of CSSA and Ap-CSSA copolymer micelles reached 600 μg/mL. Both blank micelles exhibited neglectable cytotoxicity to bEnd.3 cells and U87 MG cells. However, the cell cytotoxicity of DOX-loaded nanoparticles increased markedly. Result in Figure 1(E) indicated that the IC_50_ value was 0.83 ± 0.11 μg/mL for Ap-CSSA/DOX nanoparticles, 2.32 ± 0.08 μg/mL for CSSA/DOX nanoparticles and 0.26 ± 0.04 μg/mL for DOX against U87 MG cells. Compared with CSSA/DOX nanoparticles, the antitumor activities of Ap-CSSA/DOX nanoparticles increased by more than 1.8-fold, which was mainly due to the cell targeting and fast internalization of Ap-CSSA/DOX nanoparticles in U87 MG cells with the modification of Ap.

### Cellular targeting uptake of Ap-CSSA copolymer micelles

**Figure 2.**
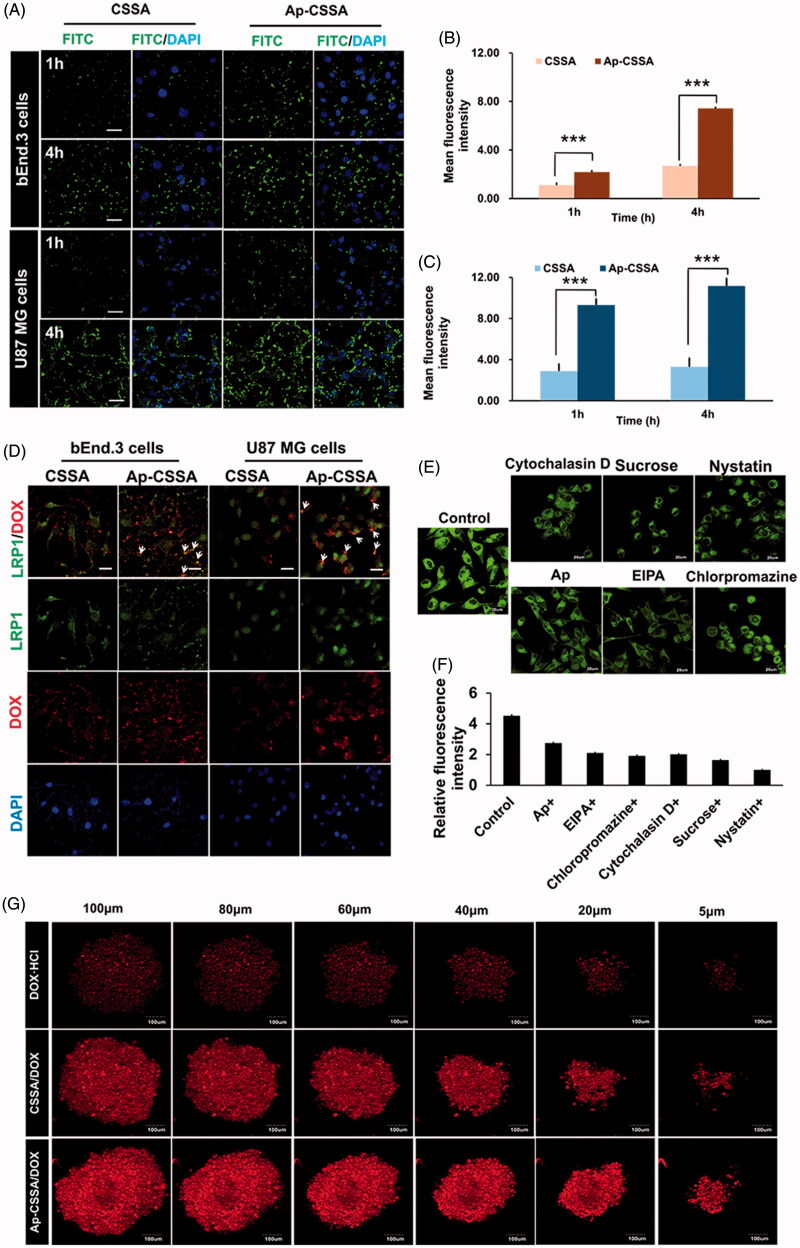
Cellular targeting uptake. (A) Intracellular fluorescence distribution in bEnd.3 cells and U87 MG cells after incubated with FITC-labeled CSSA and Ap-CSSA copolymer micelles for 1 h and 4 h. The nuclei were stained with DAPI (blue) (Scale bar =20 μm). Mean fluorescence intensity of FITC-labeled vehicles uptake in bEnd.3 cells (B) and U87 MG cells (C) measured by flow cytometer (*n* = 3). (D) *In vitro* colocalization of CSSA/DOX nanoparticles, Ap-CSSA/DOX nanoparticles with LRP1 in bEnd.3 cells and U87 MG cells. The white arrows show co-localized (yellow) of DOX-loaded nanoparticles (red) and LRP1 receptors (green) (Scale bar =30 μm). (E) The effect of different endocytosis inhibitors on Ap-CSSA copolymer micelles treated U87 MG cells (F) and relative fluorescence intensities revealing the extent of cellular uptake in each studied group. (G) Total fluorescence intensity of DOX·HCl, CSSA/DOX nanoparticles and Ap-CSSA/DOX nanoparticles on U87 MG tumor spheroid for 1 h at certain depths.

FITC-labeled Ap-CSSA copolymer micelles were used to investigate their cellular targeting capacity. Confocal images showed that the cellular uptake of CSSA and Ap-CSSA copolymer micelles in both bEnd.3 and U87 MG cells exhibited a time-dependent mode (Figure 2(A)). The cellular uptake fluorescence intensity of Ap-CSSA copolymer micelles measured by flow cytometer was significantly higher than CSSA at certain time both in bEnd.3 cells (Figure 2(B)) and U87 MG cells (Figure 2(C)). The results verified that Ap exhibited a desirable cellular intake enhancement in both bEnd.3 cells and U87 MG cells, which shared high-density LRP1 expression.

### Internalization mechanism of Ap-CSSA copolymer micelles

It has been demonstrated that both BBB and GBM cells highly expressed LPR1, here the transcytosis process of Ap-CSSA/DOX nanoparticles were evaluated. As shown in Figure 2(D), the yellow fluorescence indicated colocalization of DOX (red) and LRP1 (green), and its distribution in Ap-CSSA/DOX nanoparticles treated bEnd.3 and U87 MG cells was obviously stronger than CSSA/DOX, suggesting cellular uptake of Ap-CSSA/DOX nanoparticles was mediated by LRP1. With free Ap peptide saturated in advance, cellular uptake of Ap-CSSA copolymer micelles in U87 MG cells evidently reduced (Figure 2(E)), further indicating that the cell internalization of Ap-CSSA copolymer micelles was LRP1 receptor-mediated. Furthermore, chlorpromazine hydrochloride and sucrose were considered as clathrin-mediated pathway inhibitors, cytochalasin and EIPA as macropinocytosis pathway inhibitors, nystatin as caveolae-mediated pathway inhibitor. The fluorescence intensity was dramatically impaired by additional chlorpromazine hydrochloride, sucrose, cytochalasin D, EIPA and nystatin (Figure 2(E, F)), which indicated that the endocytic pathway of Ap-CSSA copolymer micelles in U87 MG cells also included caveolae-mediated pathway, clathrin-mediated pathway and macropinocytosis pathway.

### Spheroids uptake of Ap-CSSA/DOX nanoparticles

Solid tumor shares several barriers, including increased interstitial pressure, the absence of vascular and high cell density, which prevents lots of chemotherapeutics from delivering deeper into the core regions (Zong et al., 2014). Here, we found that Ap possessed a high LRP1-mediated cellular uptake capability in the three-dimensional U87 MG spheroids as we supposed. Compared with DOX·HCl and CSSA/DOX nanoparticles, Ap-CSSA/DOX nanoparticles displayed the strongest fluorescence signal at every regular depth of tumor spheroids (Figure 2(G)). Total fluorescence intensity of Ap-CSSA/DOX nanoparticles exhibited 1.4-fold that of CSSA/DOX nanoparticles and almost 3.8-fold higher than that of DOX·HCl (Figure S2).

### BBB pathological fenestration *in vitro*

**Figure 3.**
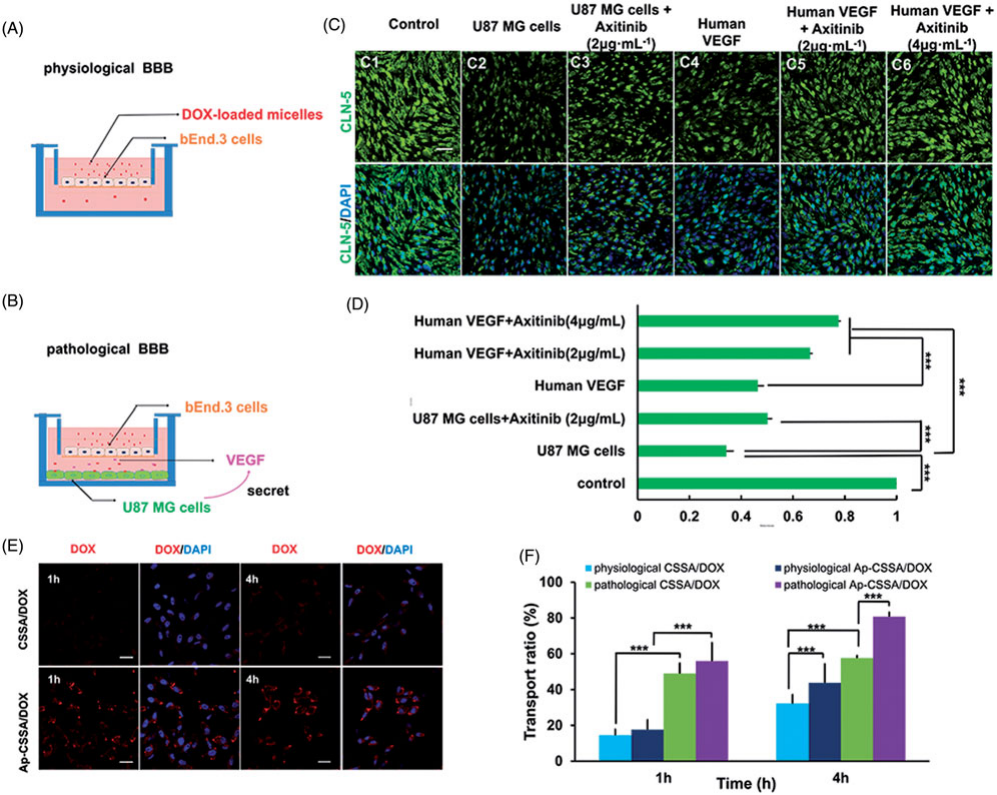
BBB pathological leakage provided an effective pathway for Ap-CSSA/DOX nanoparticles crossing the barrier and GBM cascade-targeting *in vitro*. An *in vitro* physiological BBB model (A) and pathological BBB model (B). (C) Effect of VEGF/VEGFR signal pathway on claudin-5 expression in the bEnd.3 cells observed by confocal imaging (Scale bar =30 μm). (D) The fluorescence intensity of claudin-5 signal measured by Image J (*n* = 3). (E) Cascade-targeting capacity of CSSA/DOX and Ap-CSSA/DOX nanoparticles in U87 MG cells at pathological BBB model (Scale bar =30 μm). (F) The transport ratios of CSSA/DOX and Ap-CSSA/DOX nanoparticles across the physiological BBB mode and pathological BBB model *in vitro* over a period of 1 h, 4 h (*n* = 3).

In GBM, TJs between vascular endothelial cells characteristically lose their integrity properties, which results in BBB fenestration. Studies showed that claudin-5 played a key role in TJs performance and BBB properties and claudin-5 was usually considered as a marker of BBB disruption (Ohtsuki et al., 2007). To analyze pathological breakdown of the BBB, an *in vitro* pathological BBB model (Figure 3(B)) and a physiological BBB model (Figure 3(A)) were established for evaluation. bEnd.3 cells covered on polycarbonate membranes were immune-stained for claudin-5. As showed in Figure 3(C), immunoreactivity for claudin-5 appeared discontinuous and patchy, and the fluorescent intensity was obviously weak in pathological BBB model (Figure 3(C2)). In contrast, integrity barrier was observed and claudin-5 staining appeared continuous and smooth in physiological BBB model (Figure 3(C1)).

**Figure 4.**
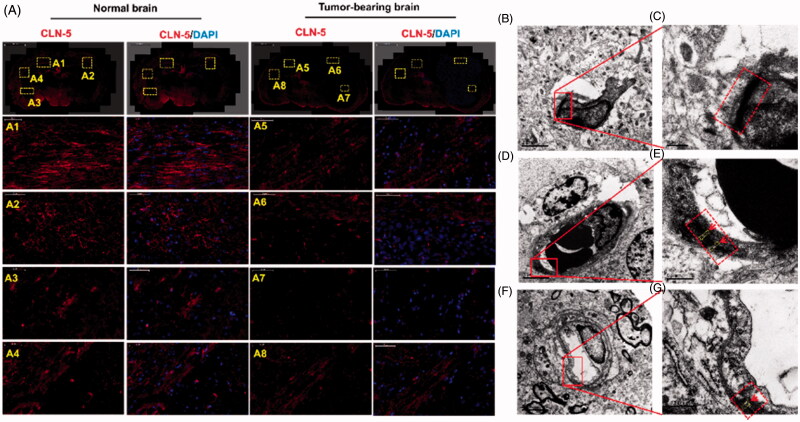
Disruption of TJs led to BBB breakdown, together with an increased bio-distribution of Ap-CSSA *in vivo*. (A) Confocal *x*–*y*–*z* scanning images of normal brain tissue section (A1–A4) and GBM-bearing brain (A5–A8), immune-stained for claudin-5 (red) and nuclear (blue) (Scale bar =50 μm). Ultrastructure of TJs in normal brain tissues (B, C), brain tumor (D, E) and the margin tissues of brain tumor (F, G) by TEM.

Since VEGF is an important factor in destructing the BBB in autoimmune encephalomyelitis (Argaw et al., 2009) and leukemia (Feng et al., 2011), we wondered whether VEGF secreted by GBM cells was also involved in the molecular regulatory mechanism of BBB pathological leakage. Therefore, ectogenic VEGF and its signaling inhibitor anxitinib (Siedlecki et al., 2017) were used to further verify our hypothesis. When axitinib was added into the pathological BBB model, the expression of claudin-5 was up-regulated (Figure 3(C2, C3)), but still lower than the normal BBB (Figure 3(C1)). On the other hand, human VEGF and axitinib with different concentration (0, 2, 4 μg/mL) were added into the physiological BBB model. Compared with normal BBB (Figure 3(C1)), we saw that the expression of claudin-5 weaken with extra human VEGF (Figure 3(C4)), while its immune-reactivity increased with additive axitinib (Figure 3(C5, C6)). The fluorescence intensity of claudin-5 signal measured by Image J was showed in Figure 3(D). The phenomenon was consistent with our hypothesis that VEGF secreted by GBM was involved in the regulation of BBB integrity. The expression of TJs and BBB pathological fenestration *in vivo* was further evaluated by transmission electron microscopy (TEM) (Figure 4(B–G)).

GBM growth disrupted the expression of TJ protein claudin-5 and thus induced BBB collapsed. The leakage of BBB provided an effective penetration pathway for Ap-CSSA/DOX nanoparticles crossing the BBB, which was an explanation for the prominent enhancement of transmembrane transport of DOX-loaded micelles in pathological BBB shown in Figure 3(E, F).

### Transportation of Ap-CSSA/DOX nanoparticles crossing pathological BBB and physiological BBB *in vitro*

The two-order cellular uptake imaging of CSSA/DOX and Ap-CSSA/DOX nanoparticles in U87 MG cells on pathological BBB model were exhibited in Figure 3(E). At predetermined time, red fluorescent intensity of Ap-CSSA/DOX nanoparticles was obvious higher than that of CSSA/DOX nanoparticles. The result further verified that Ap shared a significant potency for dual-targeting. Result in Figure 3(F) showed that the cumulative transmembrane transport ratio of Ap-CSSA/DOX nanoparticles across the pathological BBB model at 4 h was 80.8%, and that in physiological BBB model was 43.7%, which showed nearly 3.9-fold and 1.2-fold that of CSSA/DOX nanoparticles, respectively. Interestingly, the accumulation transmembrane transport ratio of Ap-CSSA/DOX nanoparticles ranged from 2.5-fold to 3.8-fold that of CSSA/DOX nanoparticles in physiological BBB model. One reason was that Ap modification enhanced both BBB traverse efficiency and two-order brain tumor targeting effect of Ap-CSSA/DOX nanoparticles. The other could result from BBB pathological leakage.

### BBB pathological disruption *in vivo*

Orthotopic brain tumor models were established for *in vivo* study (Liu et al., 2014; Miao et al., 2014). We observed that the fluorescence intensity of claudin-5 in normal brain (Figure 4(A1–A3)) was distinctly higher than that of tumor-bearing brain (Figure 4(A5–A7)), and claudin-5 in tumor-bearing brain was discontinuous and not compact. The phenomenon was conformed to the result observed *in vitro* (Figure 3(C1-C2)). TEM was used to investigate the ultra-structure changes of BBB in normal mice and tumor-bearing mice. We observed that the TJs appeared discrete and discontinuous in tumor regions (Figure 4(D,E)) and margin regions (Figure 4(F, G)) of brain tumor. Inversely, the TJs were integrated and continued (Figure 4(B,C)) in normal brain. The results indicated that GBM growth in the brain seriously affected and disrupted the ultra-structure of TJs *in vivo*, thus leading to the pathological leakage of the BBB and the increase of BBB permeability.

### Bio-distribution of Ap-CSSA traversing pathological BBB and physiological BBB *in vivo*

**Figure 5.**
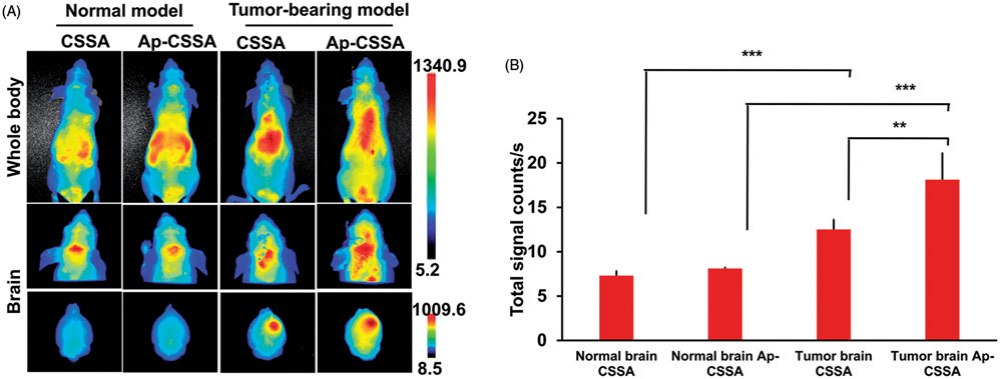
(A) *In vivo* biodistribution of CSSA/DiR, Ap-CSSA/DiR on normal and tumor-bearing nude mice after *i.v* injection and (B) the signal counts analysis (*n* = 3).

**Scheme 1.**
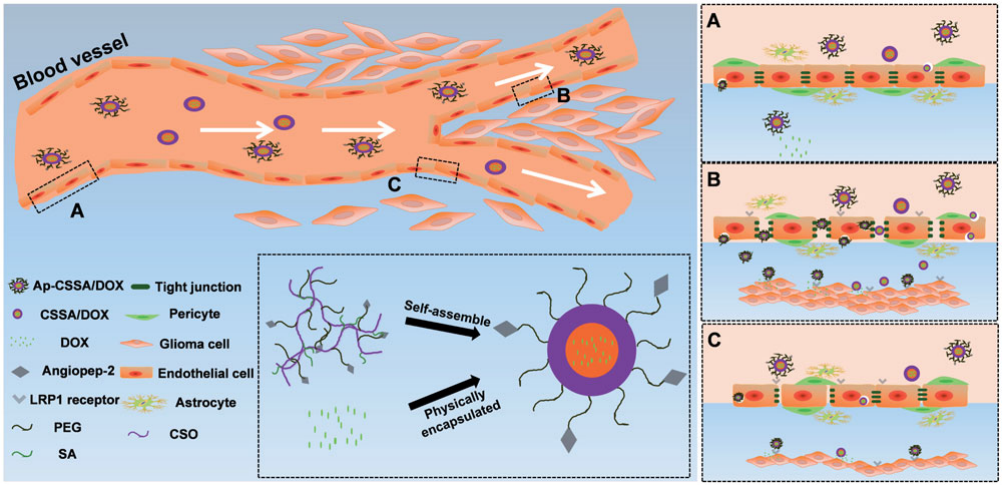
Transport routes of Ap-CSSO/DOX nanoparticles crossing the BBB. (A) Normal BBB with integrated TJs. (B) TJs was completely broken in GBM tissues. (C) The margin between normal brain tissues and GBM, TJs was slightly disrupted.

We found that the leakage of BBB provided an effective pathway for Ap-CSSA/DOX nanoparticles crossing the barrier and then cascade-targeting GBM cells (Figure 3(F)). Herein, we wondered if the same phenomenon would happen *in vivo*. Figure 5(A) showed the biodistribution of CSSA/DiR and Ap-CSSA/DiR in normal nude mice and tumor-bearing nude mice, the fluorescence intensity of Ap-CSSA/DiR group in brain was higher than that of CSSA/DiR in both normal and tumor-bearing model. The results were mainly due to Ap-targeting capacity. And interestingly, the bio-distribution of Ap-CSSA/DiR in tumor-bearing brain was significantly stronger than normal brain (Figure 5(A)), and the signal counts in tumor-bearing brain was nearly 2.2-fold that of normal brain (Figure 5(B)). This results might be on account of that the BBB in normal nude mice was integrated and the permeability was weak. However, with the growth of GBM cells and deterioration of pathological degree, the permeability of BBB increased. Also the number of LRP1 in brain increased rapidly, and a higher density distribution of LRP1 were observed in tumor-bearing brain tissues (Figure S3), further exposing more LRP1 binding sites for Ap-CSSA/DiR. Under pathological condition, Ap-CSSA/DiR crossed the BBB not only by LRP1-mediated transcytosis pathway, but also by an extra BBB-opening pathway for GBM cascade-targeting (Scheme 1(B)). Therefore, the Ap-CSSA/DiR reached the brain tumor rapidly and the fluorescence intensity was stronger in tumor-bearing brain than normal brain.

### *In vivo* competition biodistribution of Ap-CSSA

A competition assay was used to verify the *in vivo* transportation pathway of Ap-CSSA copolymer micelles. Because of the saturation effect with free Ap peptide in advance, the bio-distribution of Ap-CSSA/DiR in brain tumor strictly decreased at certain time (Figure S4A), and the fluorescence signals of dissected tumor-bearing brain tissues were also observed (Figure S4B). Semiquantitive fluorescence signals were shown in Figure S4C. The total signal counts on brain tumor decreased 2.2-fold with an additive Ap injection beforehand. The result verified that the bio-distribution of Ap-CSSA copolymer micelles *in vivo* was also LRP1 mediated.

### *In vivo* antitumor activity

**Figure 6.**
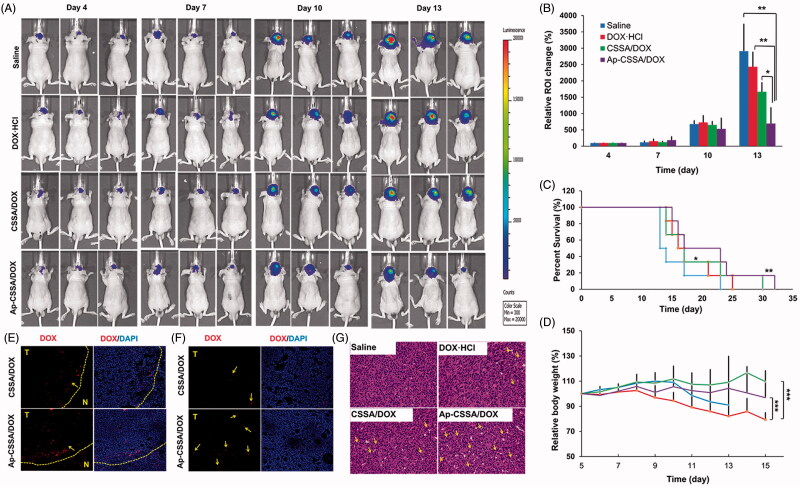
Antitumor activity. (A) *In vivo* bioluminescence imaging of U87-luci bearing nude mice at different time points after treatment (day 4, 7, 10, 13). (B) Quantitative of signals from the entire abdominal region of each mouse (*n* = 3). (C) Kaplan–Meier survival curves (*n* = 6). (D) Body weight changes exposed to various treatment (*n* = 6). *In vivo* distribution of DOX-loaded nanoparticles in the margin (E) and center areas (F) of the orthotopic brain tumor. (G) H and E staining of brain tumor sections and heart sections after the *i.v.* injection with saline, DOX·HCl, CSSA/DOX and Ap-CSSA/DOX nanoparticles, respectively.

Because of the specific growth site of the GBM, we used bioluminescence imaging to monitor tumor growth response in a real-time and noninvasive way (Li et al., 2015), which was actively employed to study cancer treatment such as metastasis of lungs (Taromi et al., 2016) and lymph (Mollard et al., 2016). The bioluminescent intensity of U87-luci cells which express luciferase could be used to quantify the survival of the tumor cells. Tumor-bearing mice were administered with saline, DOX·HCl, CSSA/DOX nanoparticles and Ap-CSSA/DOX nanoparticles, respectively. The bioluminescence imaging and signal intensity of different formulations groups were summarized in Figure 6(A). All groups were administered at a multidose of 2 mg DOX/kg via the tail vein on day 5, 8 and 11 postinoculation. We could found that the bioluminescence imaging signals of Ap-CSSA/DOX group were clearly lower than other groups at 2 days after every injection (Figure 6(A)). The average bioluminescence intensity changes in the ROI of saline, DOX·HCl, CSSA/DOX, Ap-CSSA/DOX groups were 2912.7%, 2433.0%, 1667.7% and 696.7% on day 13, respectively (Figure 6(B)). The median survival times of saline, DOX·HCl, CSOSA/DOX nanoparticles and Ap-CSSA/DOX nanoparticles treated tumor-bearing nude mice were 13.5, 16.5, 17.0 and 23.0 days, respectively (Figure 6(C)). Compared with other DOX formulations and saline group, Ap-CSSA/DOX group showed a significant prolonged survival span of tumor-bearing mice. Notably, the body weight of DOX·HCl group decreased significantly and caused over 10% of body weight loss on average during the treatments (Figure 6(D)). No notable changes were found in Ap-CSSA/DOX group and the other groups. Furthermore, a significantly higher distribution of Ap-CSSA/DOX nanoparticles was observed in the tumor (Figure 6(E,F)). All these results provided robust evidence for the hypothesis that Ap might serve as an effective targeting capacity in brain tumor therapy.

The histopathologic features of tumors and hearts from mice in all treatment groups were examined by H and E staining. As shown in Figure 6(G), there was a remarkable greatest amounts of necrosis (yellow arrows) detected in Ap-CSSA/DOX group, and smaller amounts were observed in DOX·HCl group, CSSA/DOX group and saline group. The notable cell lethal effect of Ap-CSSA/DOX group was mainly attributed to enhanced brain transport by pathological opening of BBB, as well as promoted intracellular delivery by Ap modification. In addition, myocardial hypertrophy was shown clearly in the heart section of DOX·HCl group (Figure S5), while no evidence of abnormal morphological features was found in Ap-CSSA/DOX group and other formulations groups. These results were also demonstrated that Ap-CSSA/DOX could deliver antitumor drugs into GBM cells efficiently and serve as a safe drug delivery.

## Discussion

The integrity of BBB seriously restricts the distribution of chemotherapeutics from blood to brain and brain tumor, and GBM predicts very poor prognosis (Qin et al., 2017). So the development of novel drug delivery systems that can transport drugs across the BBB and target brain tumor regions is urgently needed. In this study, we found that the growth of GBM resulted in the pathological fenestration of TJs and the pathological opening of BBB (Figures 3(C) and 4). Since both BBB and GBM exhibited high expression of LRP1 (Warshawsky et al., 1994), and with the deterioration of pathological degree, large amounts tumor cells with a higher density distribution of LRP1 were found in brain (Figure S3). BBB pathological fenestration exposed more LRP1-binding sites for its specific ligands, also provided more opportunities for TNDDS to distribute in brain (Scheme 1(A,B)). Since Ap exhibited high affinity to LRP1, here we used Ap to decorate on the surface of glycolipid-like nanoparticles (Ap-CSSA/DOX) for BBB targeting and brain tumor cascade-targeting, thus realizing GBM effective therapy.

However, a large number of researches confirmed that TNDDS traversed the BBB mainly by receptor-mediated pathway (Ying et al., 2010; Huang et al., 2011a), but with little investigation between the pathological fenestration of BBB and the transportation of TNDDS. Besides, the pathological changes of BBB was involved in many CNS disease (Kirk et al., 2003) (Claudio, 1995), but its pathogenesis in GBM and its pathological fenestration in regulating TNDDS transport across the barrier were poorly understood. Here, we constructed a physiological BBB model (Figure 3(A)) and a pathological BBB model (Figure 3(B)) *in vitro*. And we found that VEGF secreted by U87 MG cells seriously decreased the expression of TJs protein claudin-5 (Figure 3(C2)), inhibition of VEGF/VEGFR signal pathway upregulated claudin-5 and reduce the permeability of the BBB (Figure 3(C3–C6)). In addition, results showed that down-regulation of claudin-5 in pathological BBB model significantly increased the transport of Ap-CSSO/DOX nanoparticles crossing the barrier (Figure 3(F)) and then for GBM cascade-targeting uptake (Figure 3(E)). BBB pathological opening also significantly enhanced the bio-distribution of Ap-CSSA copolymer micelles in brain tumor (Figure 5) *in vivo*.

With the contribution of BBB pathological fenestration and Ap modification, Ap-CSSA/DOX nanoparticles exhibited a considerable tumor-targeting penetration distribution capacity (Figure 6(E,F)) and an appreciable anti-tumor effect *in vivo* (Figure 6(A–D)). Ap-CSSA//DOX nanoparticles also displayed as a safe TNDDS (Figures 6(D) and S5).

## Conclusions

In this study, we presented a DOX-loaded targeting glycolipid-like drug delivery system (Ap-CSSA/DOX), exhibiting high affinity to LRP1 over-expressed on both BBB and GBM cells. GBM growth resulted in TJs disruption and BBB pathological breakdown, which provided an extra effective pathway for Ap-CSSA/DOX nanoparticles crossing the BBB. A significantly enhanced cascade-targeting and bio-distribution of Ap-CSSA/DOX nanoparticles in tumor region ascribed to both Ap decoration and BBB pathological leakage, finally produced an observable anti-tumor effect *in vivo*. This might also provide a new sight in other CNS diseases treatment.

## Supplementary Material

IDRD_Hu_et_al_Supplemental_Content.docx
