# Supplementary material for: VEGF-mediated tight junctions pathological fenestration enhances doxorubicin-loaded glycolipid-like nanoparticles traversing BBB for glioblastoma-targeting therapy
Source: Drug Deliv. 2017 Nov 28;24(1):1843–55. doi: 10.1080/10717544.2017.1386731 (PMC8241127; doi:10.1080/10717544.2017.1386731)
Supplement: IDRD_Hu_et_al_Supplemental_Content.docx [file IDRD_A_1386731_SM7101.docx]

**Supporting Information**

**VEGF mediated Tight Junctions Pathological Fenestration Enhances Doxorubicin Loaded Glycolipid-like Nanoparticles Traversing BBB for Glioblastoma Targeting Therapy**

Lijuan Wen ^1^, Yanan Tan ^2^, Suhuan Dai ^1^, Yun Zhu ^2^, Tingting Meng ^1^, Xiqin Yang ^1^ Yupeng Liu ^1^, Xuan Liu ^1^, Hong Yuan ^1^, and Fuqiang Hu ^1*^

^1^ College of Pharmaceutical Science, Zhejiang University, 866 Yuhangtang Road, Hangzhou 310058, People’s Republic of China.

^2^ Ocean College, Zhejiang University, Zheda Road, Zhoushan 316021, People’s Republic of China.

*Correspondence author: Fuqiang Hu, College of Pharmaceutical Science, Zhejiang University, 866 Yuhangtang Road, Hangzhou 310058, People’s Republic of China. Tel/Fax: +86-571-88208439. E-mail: [hufq@zju.edu.cn](mailto:hufq@zju.edu.cn).

**Table S1.** Characteristics of blank copolymer micelles and DOX-loaded nanoparticles.


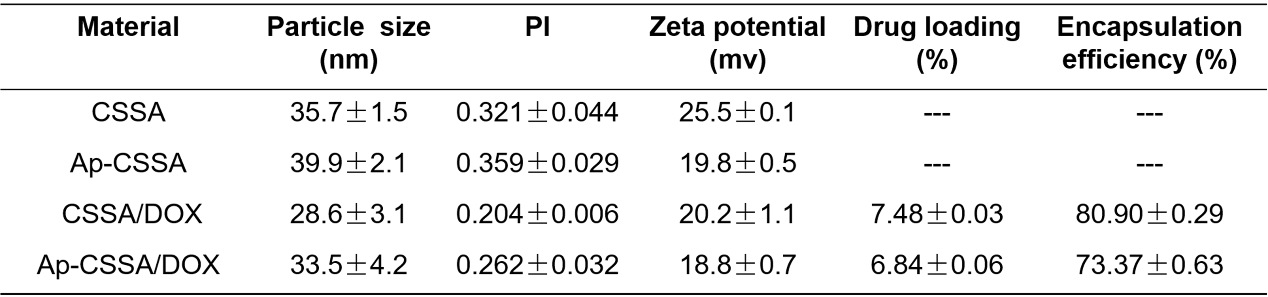


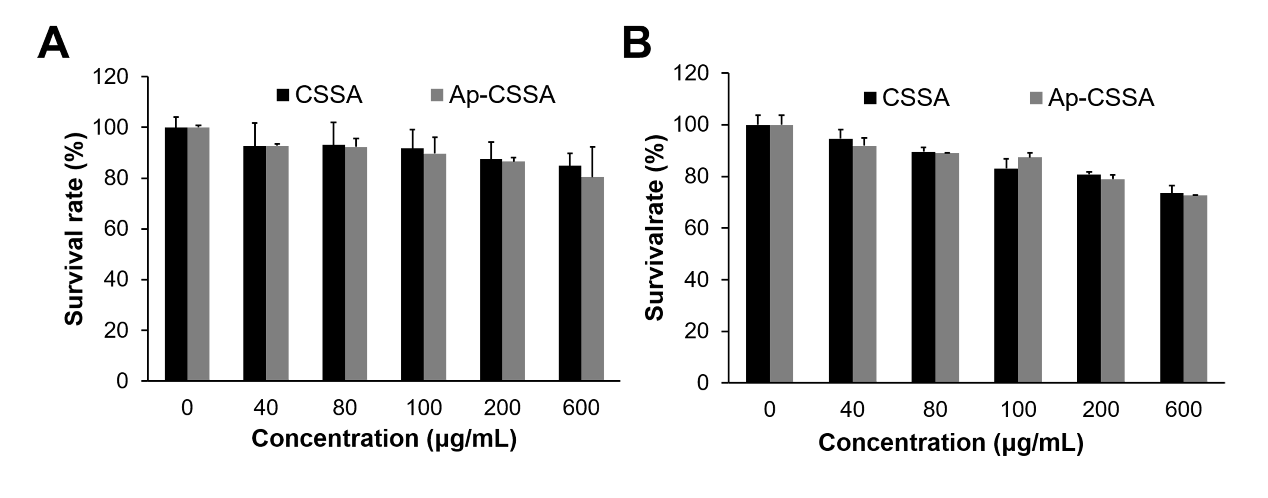


**Figure S1. Cell cytotoxicity of blank vehicles.** *In vitro* cell viability of bEnd.3 cells (**A**) and U87 MG cells (**B**) after treated with CSSA and Ap-CSSA copolymer micelles (n=5).


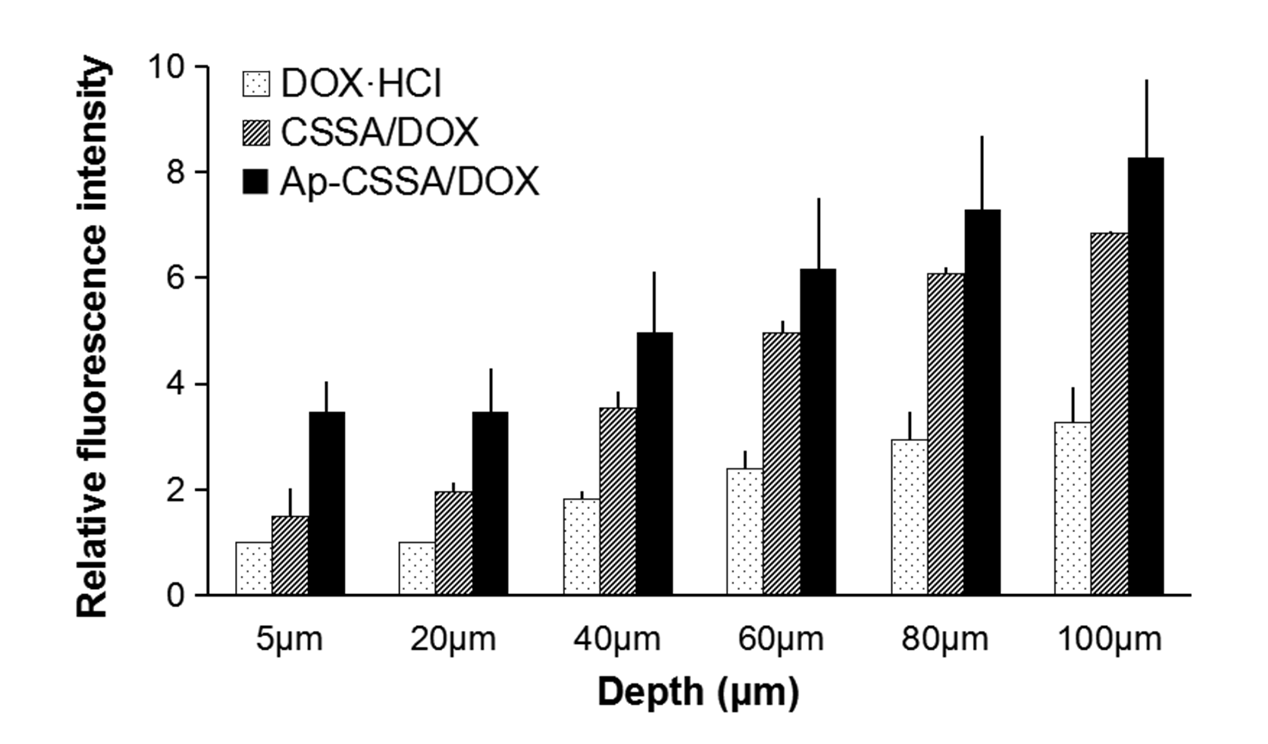


**Figure S2: Fluorescence intensity analysis.** Merged fluorescence intensity (red channel) at every regular depth of U87 MG spheroids quantified from CLSM (n=3).

**
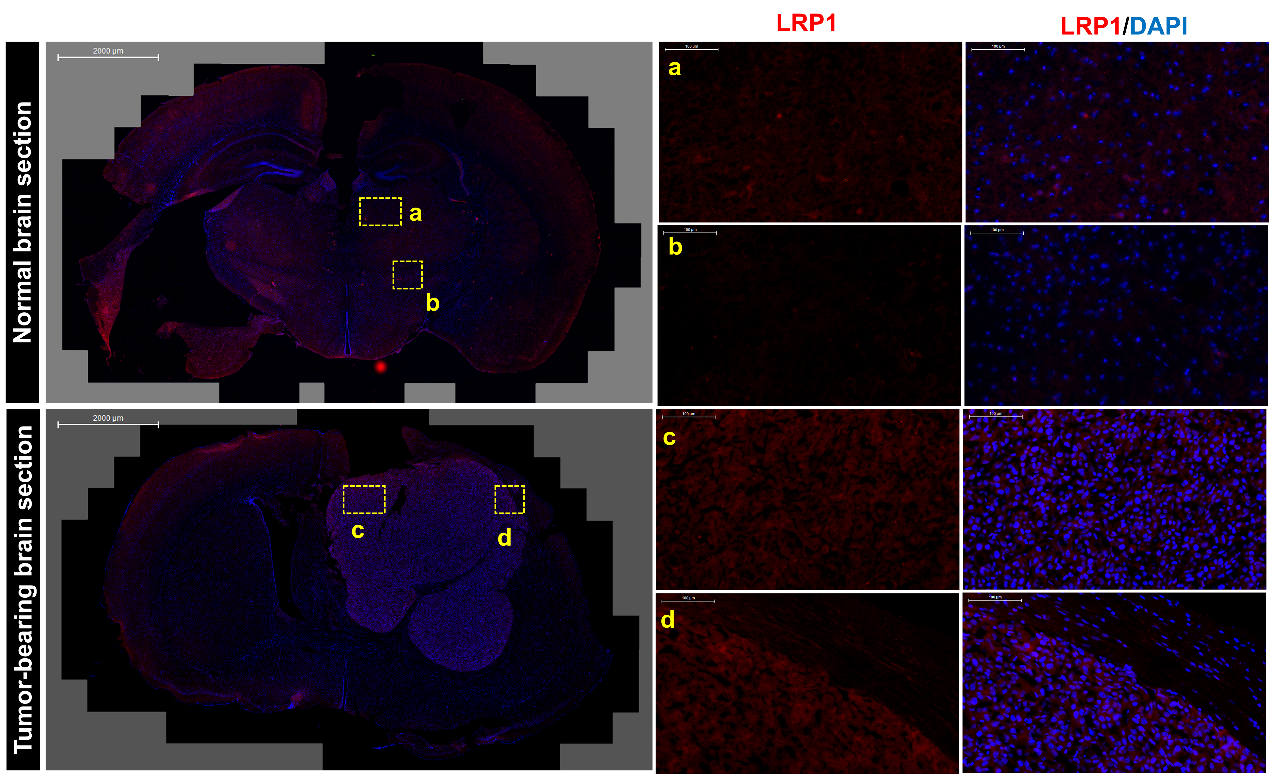
**

**Figure S3: LRP1 expression.** LRP1 expression in normal brain sections (**a, b**) and tumor-bearing brain sections (**c, d**). (Scale bar = 200 μm)


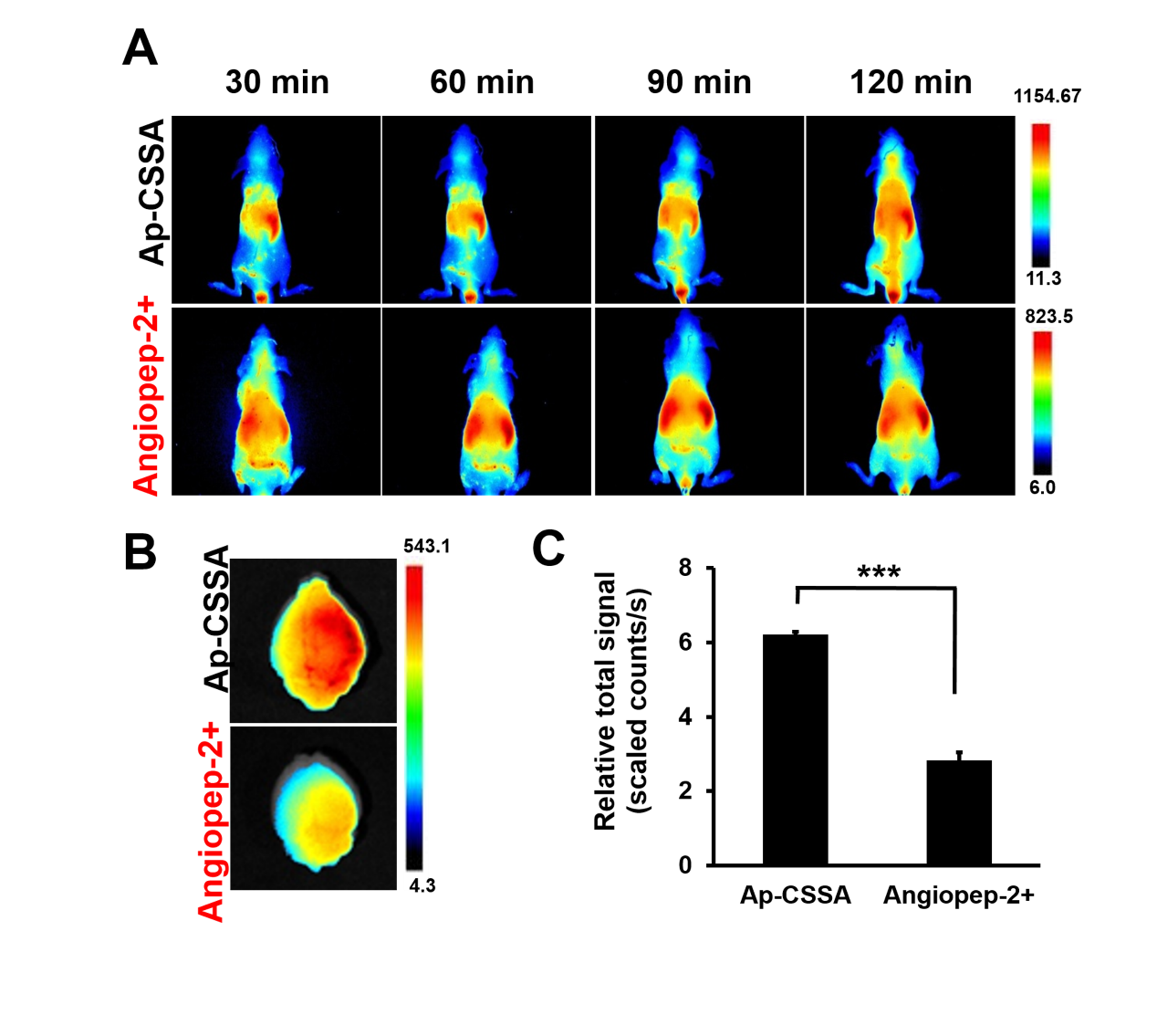


**Figure S4. *In vivo* competition bio-distribution of Ap-CSSA copolymer micelles.** Fluorescence distribution of Ap-CSSA/DiR with or without additive Ap injection in advance in whole body (**A**) and excised brain (**B**). (**C**) Bio-distribution signal analysis of dissected brain tissues (n=3).


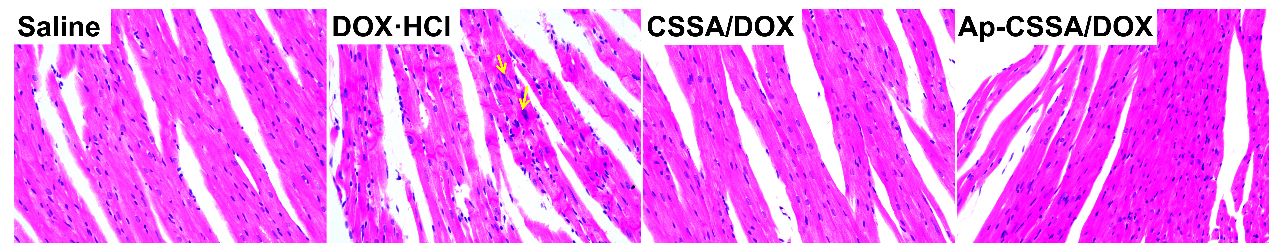


**Figure S5.** H&E staining of heart sections after the i.v. injection of saline, DOX·HCl, CSSA/DOX and Ap-CSSA/DOX nanoparticles, respectively.
